# Multitrait Screening and SSR Fingerprinting of *Calligonum* Section *Medusa* Individuals for Desert Ecological Restoration

**DOI:** 10.3390/plants15203169

**Published:** 2026-10-16

**Authors:** Mingzhi Du, Yinjuan Yao, Zhongchen Lu, Jing Zhang, Xiyong Wang, Jing Zhang, Jiancheng Wang, Wei Shi

**Affiliations:** 1 College of Forestry and Landscape Architecture, Xinjiang Agricultural University, Urumqi 830052, China; d1017486108@163.com (M.D.); 15099517507@163.com (Y.Y.); zhangjing20242944@163.com (J.Z.); 2 State Key Laboratory of Desert and Oasis Ecology, Key Laboratory of Ecological Safety and Sustainable Development in Arid Lands, Xinjiang Institute of Ecology and Geography, Chinese Academy of Sciences, Urumqi 830011, China; 17630281421@163.com (Z.L.); wangxy@ms.xjb.ac.cn (X.W.); zhangjing@ms.xjb.ac.cn (J.Z.); 3 Turpan Eremophytes Botanical Garden, Xinjiang Institute of Ecology and Geography, Chinese Academy of Sciences, Turpan 838008, China; 4 China-Tajikistan Belt and Road Joint Laboratory on Biodiversity Conservation and Sustainable Use, Xinjiang Institute of Ecology and Geography, Chinese Academy of Sciences, Urumqi 830011, China

**Keywords:** *Calligonum* sect. *Medusa*, desert ecological restoration, multitrait evaluation, candidate elite individuals, SSR fingerprinting

## Abstract

*Calligonum* sect. *Medusa* is an important resource for desert ecological restoration, but practical criteria for elite germplasm screening and reliable methods for identity verification remain limited. We evaluated 13 morphological, physiological, and biochemical traits in 121 individuals conserved ex situ at the Turpan Campus of Yili-Tupan National Botanical Garden (electable) using principal component analysis, membership-function evaluation, hierarchical clustering, and stepwise regression; 20 individuals were further genotyped at 13 simple sequence repeat (SSR) loci. Trait coefficients of variation ranged from 6.54% to 53.79%, and five principal components explained 68.40% of the total variation. Five candidate elite individuals (ZLY37, ZLY40, ZLY44, ZLY38, and ZLY01) were identified. A reduced six-trait model retained crown width, predawn water potential, plant height, soluble sugar content, plant-form integrity, and assimilating-branch length, from which preliminary empirical reference thresholds were derived. Of the 13 SSR loci, 10 were polymorphic (mean PIC = 0.357), and a candidate five-locus panel distinguished the same 20 individuals used for its selection and enabled fingerprint construction for the five candidates. This study provides a practical framework for preliminary elite germplasm screening and molecular identity verification in *Calligonum* sect. *Medusa*, supporting desert ecological restoration, germplasm management, and subsequent cultivar development.

## 1. Introduction

The expansion of global drylands and continuing land degradation have intensified the need for effective desert ecosystem restoration [1,2]. Restoration outcomes depend not only on species choice but also on the adaptive capacity and functional traits of the planting material [3,4]. Species of *Calligonum* L. are widely used as windbreak and sand-fixing shrubs in the deserts of Central Asia and northwestern China [5,6]. Their highly reduced leaves and photosynthetic assimilating branches [7], well-developed root systems and drought tolerance [8], and crown architecture that modifies near-surface airflow [9,10] underpin their restoration value. Long-term *Calligonum* plantations can also promote vegetation recovery and increase plant–soil carbon and nitrogen pools [11]. Based primarily on fruit morphology, *Calligonum* has traditionally been divided into four sections: *Calliphysa*, *Pterococcus*, *Calligonum*, and *Medusa* [12]. Among these, sect. *Medusa* is the most species-rich and geographically widespread section in Xinjiang [13], making it an especially relevant target for germplasm evaluation and restoration-oriented selection. However, *Calligonum* taxa and individuals vary in phenology, water status, and physiological responses to stress [14,15].

Selection of elite woody plant material commonly integrates growth, crown form, physiological condition, and other target traits through multivariate analysis and comprehensive evaluation. Studies of *Pinus yunnanensis*, *Fraxinus mandshurica*, and *Tilia amurensis* illustrate the value of integrating complementary growth, form, and stress-response traits when identifying candidate elite material [16,17,18]. In *Calligonum*, previous work has examined drought and salinity responses [19,20,21] and molecular diversity [22,23]. Zhang et al. used PCA to evaluate drought-related variation across 16 species of this genus using 17 indices, including physiological and biochemical traits and estimated biomass [14]. This species-level comparison provided a basis for multivariate evaluation in *Calligonum* but did not establish reduced indicators or empirical thresholds for selecting individual plants within ex situ collections. Reliable identity verification is also needed during ex situ conservation, propagation, subsequent trials, and cultivar development. Within *Calligonum* sect. *Medusa*, the *C. mongolicum* complex illustrates the difficulty of morphological identification, as fruit characters overlap and species boundaries remain debated [24,25,26,27]. Complementing phenotypic characterization, DNA fingerprints based on simple sequence repeat (SSR) markers can provide reproducible multilocus profiles for distinguishing and tracking individual accessions [28,29].

**Figure 1:**
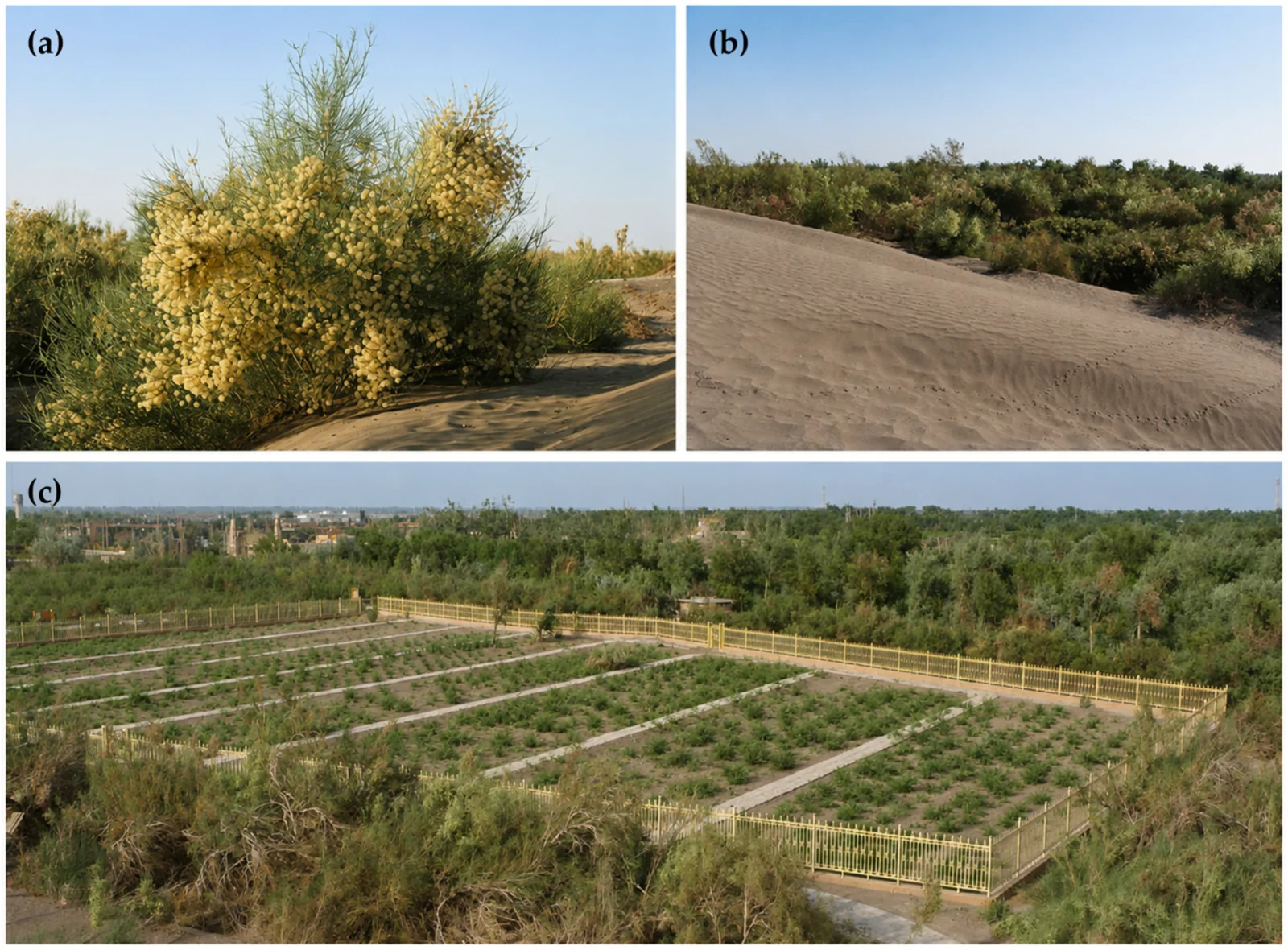
*Calligonum* sect. *Medusa* plants and their ex situ conservation areas. (**a**) A mature individual; (**b**) the *Calligonum* Specialized Garden (ZLY); and (**c**) the *Calligonum* Germplasm Resource Nursery (ZZP).

This study evaluated 121 individuals of *Calligonum* sect. *Medusa* maintained at the Turpan Campus of Yili-Tupan National Botanical Garden (electable) (Figure 1). The objectives were to (1) integrate 13 morphological, physiological, and biochemical traits to identify candidate elite individuals, derive a reduced set of screening indicators, and develop preliminary empirical thresholds for restoration-oriented screening; (2) genotype a selected subset of individuals using SSR markers and identify a reduced locus panel for individual discrimination and fingerprint construction within the current 20-individual subset. The evaluation identified five candidate elite individuals and six screening indicators with corresponding preliminary empirical thresholds, while a candidate five-locus SSR panel distinguished all 20 assayed individuals. Together, these findings link multitrait screening with molecular identity verification, providing a basis for germplasm management, subsequent cultivar development, and restoration-oriented selection.

## 2. Results

### 2.1. Variation and Correlations Among Restoration-Oriented Traits

The coefficients of variation (CVs) for the 13 traits ranged from 6.54% to 53.79%. Basal diameter (BD) had the highest CV (53.79%), followed by free proline content (Pro; 48.34%), assimilating-branch length (ABL; 38.47%), and malondialdehyde content (MDA; 38.39%). Predawn water potential (Ψpd) had the lowest CV (6.54%) (Table 1). Overall, the 13 traits exhibited varying degrees of interindividual variation, providing a basis for subsequent multivariate evaluation. Among identified species, mean PH and CW were greatest in *C. caput-medusae*, whereas mean ABL was greatest in *C. pumilum* (Table S1). ZLY had higher mean PH, CW, and ABL than ZZP. The 1977 planting group had higher mean PH and CW than both the 2011 planting group and the unresolved material with planting records spanning 1972–2013 (Table S2). The two observers assigned identical independent scores to all 121 plants for both BrD and PFI (exact agreement, 100%; linearly weighted κ = 1.000 for each trait; Table S3).

**Figure 2:**
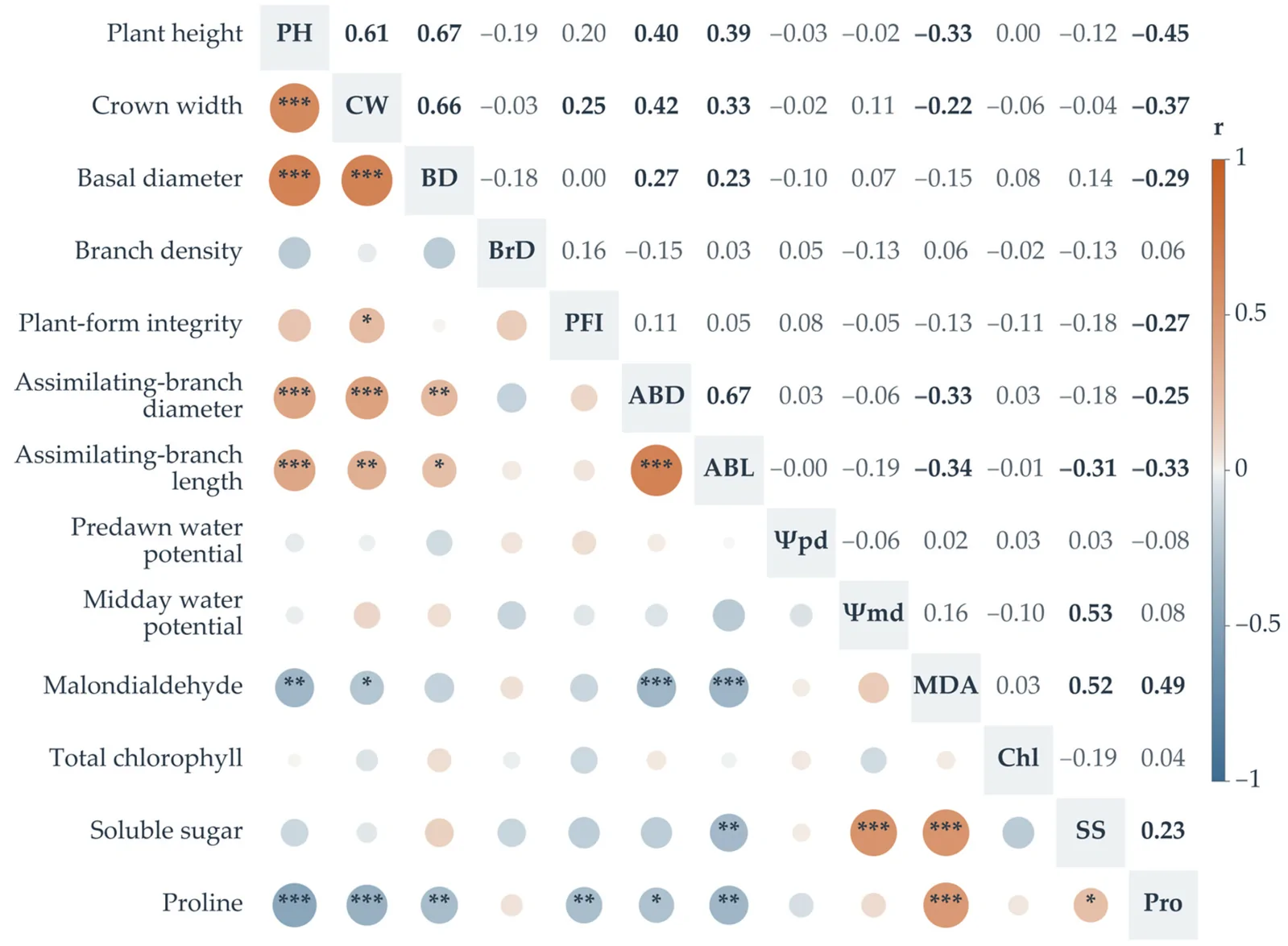
Pearson correlation matrix of the 13 restoration-oriented traits. Pearson’s correlation coefficients (r) are shown above the diagonal. Below the diagonal, circle size indicates correlation strength, while orange and blue indicate positive and negative correlations, respectively. *p* values were adjusted using the Benjamini–Hochberg procedure, with adjusted values denoted by q. Bold coefficients indicate q < 0.05. Asterisks indicate significance: * q < 0.05, ** q < 0.01, and *** q < 0.001.

The 13 traits yielded 78 unique pairwise correlations. After Benjamini–Hochberg false-discovery-rate adjustment [30], 26 correlations remained significant at q < 0.05, comprising 15 positive and 11 negative correlations (Figure 2). Positive correlations were observed between ABD and ABL (r = 0.669), PH and BD (r = 0.666), CW and BD (r = 0.658), and PH and CW (r = 0.608). Ψmd was positively correlated with SS (r = 0.527), and MDA was positively correlated with both SS (r = 0.523) and Pro (r = 0.486) (all q < 0.001). Pro was negatively correlated with PH, CW, BD, PFI, ABD, and ABL, with the strongest negative association observed between PH and Pro (r = −0.454). MDA was negatively correlated with PH, CW, ABD, and ABL, while SS was negatively correlated with ABL (all q < 0.05). Overall, several plant-size and assimilating-branch traits were positively intercorrelated, whereas MDA and Pro were negatively associated with multiple structural traits.

### 2.2. Principal Component Analysis and Selection of Candidate Elite Individuals

Principal component analysis (PCA) retained five principal components (PCs) with eigenvalues greater than one [31,32]. Their eigenvalues were 3.557, 1.978, 1.286, 1.045, and 1.026, accounting for 27.36%, 15.22%, 9.89%, 8.04%, and 7.89% of the total variance, respectively, and 68.40% cumulatively (Table 2). PC1 primarily described variation in plant size and assimilating-branch growth, with loadings of 0.793 for PH, 0.727 for CW, 0.682 for ABD, 0.676 for ABL, and 0.623 for BD. Reverse-standardized MDA loaded positively on PC1 (0.613), whereas Pro loaded negatively (−0.661). PC2 was associated mainly with SS (0.780), Ψmd (0.685), and BD (0.541). PFI, Chl, and Ψpd had the largest absolute loadings on PC3, PC4, and PC5, respectively (0.704, 0.684, and 0.644). The retained PCs therefore summarized distinct combinations of structural, biochemical, and water-status traits.

**Figure 3:**
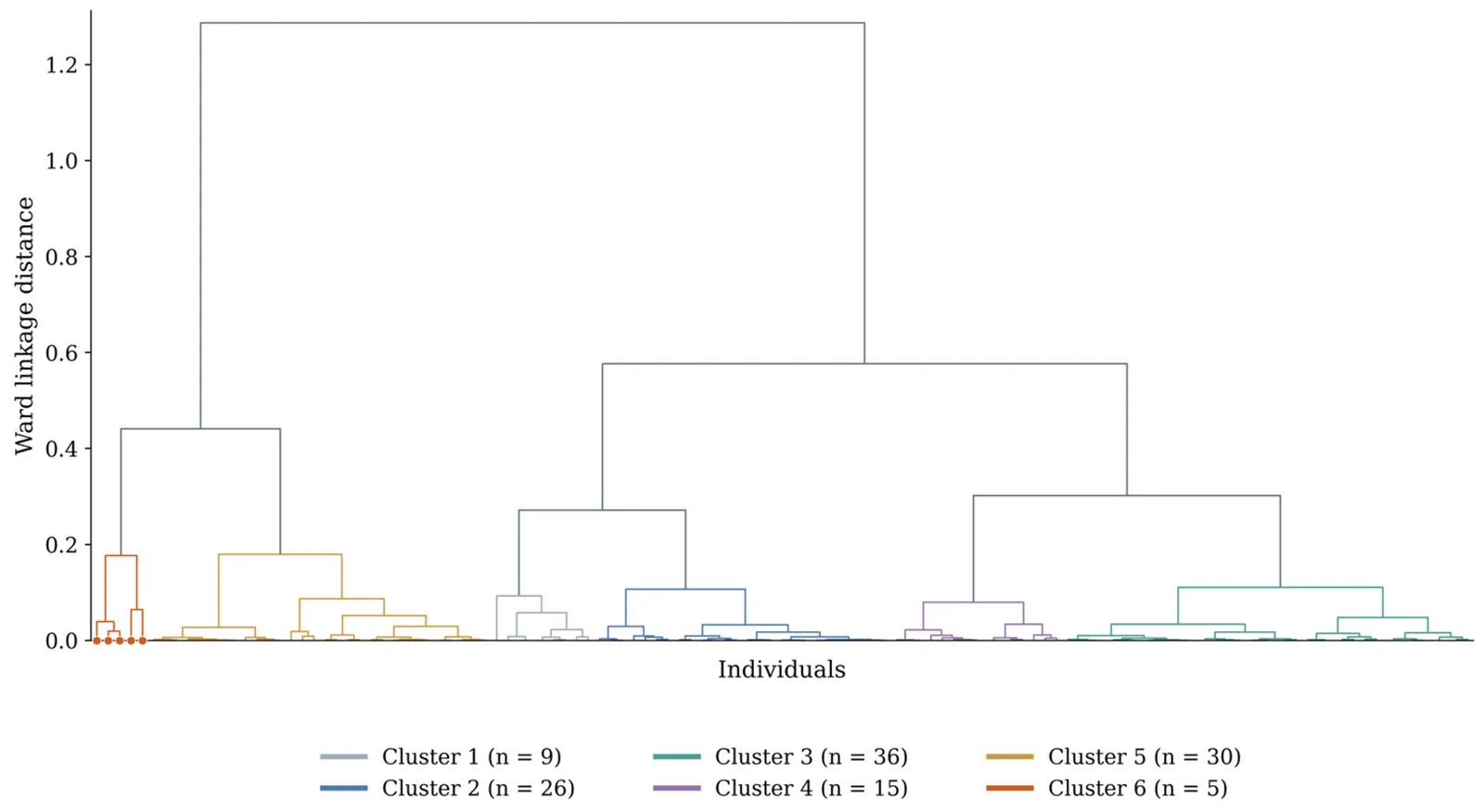
D-value hierarchical classification of the 121 individuals. Ward’s minimum-variance hierarchical clustering based on D values; different colors indicate the six groups.

**Figure 4:**
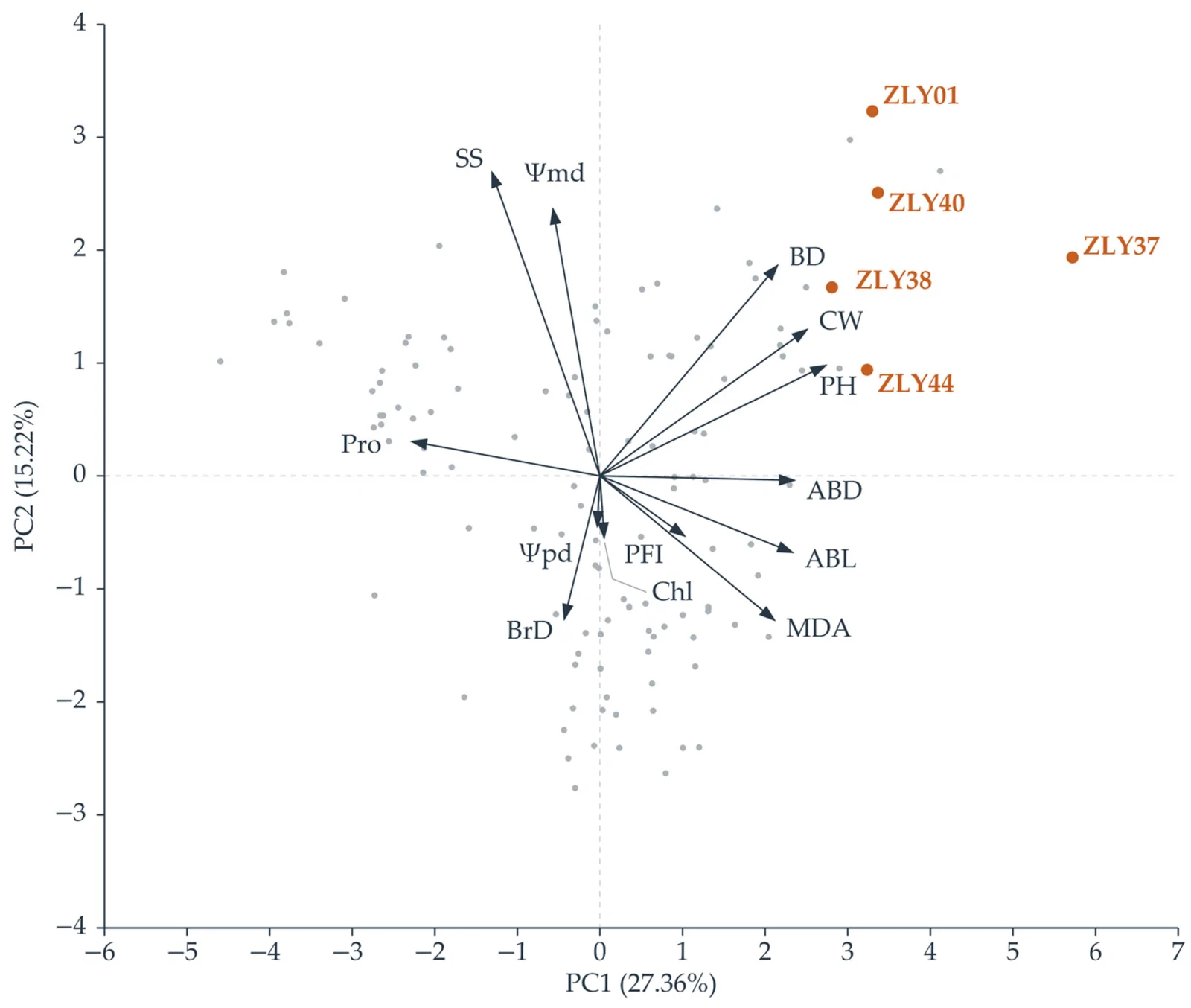
PC1–PC2 biplot of 121 individuals based on 13 directionally aligned restoration-oriented traits. Orange circles indicate the five candidate elite individuals, gray circles indicate the remaining individuals, and arrows represent trait loadings on the first two PCs.

Ward’s minimum-variance hierarchical clustering of the relative composite score (D) divided the 121 individuals into six groups (Figure 3). In ascending order of mean D, the groups contained 9, 26, 36, 15, 30, and 5 individuals. The highest-D group comprised ZLY37, ZLY40, ZLY44, ZLY38, and ZLY01, with D values of 0.826, 0.762, 0.703, 0.678, and 0.658, respectively, and a mean of 0.725 (Table S4). The same five-individual highest-D group was retained when this dendrogram was cut into four, five, six, or seven groups. These individuals also occupied the top five positions in the D ranking and were designated candidate elite individuals; all five were from ZLY. The recorded species assignments were *C. caput-medusae* for ZLY37, ZLY40, ZLY44, and ZLY38, and *C. arborescens* for ZLY01 (Table S5). PC1 and PC2 together explained 42.58% of the total variance. In the PC1–PC2 biplot, ZLY37 had the highest PC1 score, while ZLY01 and ZLY40 had relatively high PC2 scores (Figure 4). The six groups differed in their mean trait profiles (Table S6). Group 1 had the smallest mean PH and CW, the most negative mean Ψpd, and the highest mean Pro. Group 2 also had relatively small dimensions, whereas Group 3 combined intermediate dimensions with relatively high BrD. Group 4 had intermediate dimensions and the least negative mean Ψpd. Group 5 had larger dimensions and the highest mean PFI. Group 6 had the largest mean CW, BD, and ABL and the highest mean SS.

### 2.3. Reduced Screening Indicators and Preliminary Empirical Thresholds

D is a relative composite score that summarizes the 13 measured traits through the weighted membership values of the retained PCs. Stepwise regression used D as the response and the same 13 directionally standardized traits as candidate predictors. CW entered first, followed by Ψpd, PH, SS, PFI, and ABL. The five-trait model was the first to exceed R^2^ = 0.90 (R^2^ = 0.9234). Adding ABL increased R^2^ by 0.0255 to 0.9489 (adjusted R^2^ = 0.9462), whereas each subsequent addition increased R^2^ by less than 0.016. The six-trait model was retained to approximate the full D-based evaluation (Table 3). Because D was derived from the same trait set, R^2^ = 0.9489 describes how closely the model reproduces D within the fitting dataset, not independent predictive performance. CW had the largest standardized coefficient (β = 0.497), and variance inflation factors (VIFs) ranged from 1.01 to 1.71. Calculated and fitted D values were closely associated within the dataset (Pearson r = 0.974; Spearman ρ = 0.963). The reduced model recovered the same five highest-ranked individuals, although their internal order differed.

For each retained trait, the minimum value among the five candidates was used as a preliminary lower reference threshold (Table 4). When applied jointly to the 121 individuals, no additional individuals met all six thresholds within the current dataset. The PH and CW thresholds jointly retained six individuals; adding the ABL threshold excluded ZLY04, leaving the five candidates. The remaining three thresholds did not further reduce this subset. The joint PH, CW, and ABL criteria were met by four of 22 *C. caput-medusae* individuals (18.2%) and one of 19 *C. arborescens* individuals (5.3%), with no individuals from the other recorded groups meeting all three criteria (Table S1).

### 2.4. SSR Profile Variation and Relationships Among Individuals

Genotyping success across the 13 SSR loci ranged from 5.0% to 100.0%. Sgz005 yielded a valid profile in only one individual, while Sgz012 and Sgz020 were monomorphic across all 20 individuals; these three loci were excluded from clustering and fingerprint selection. The remaining 10 polymorphic loci had a mean genotyping success of 98.0%. Under the adopted two-slot profile coding, their mean Na, Ne, I, Ho, He, PIC, and DP values were 2.900, 1.979, 0.708, 0.435, 0.409, 0.357, and 0.356, respectively (Table 5). Sgz017 had the highest Na, Ne, I, Ho, He, and PIC values (5, 3.704, 1.402, 1.000, 0.730, and 0.684, respectively). Sgz001 had the highest DP (0.715) and a PIC of 0.627. Because ploidy was not independently established, these statistics describe the stored profiles and are not population-level diversity estimates.

**Figure 5:**
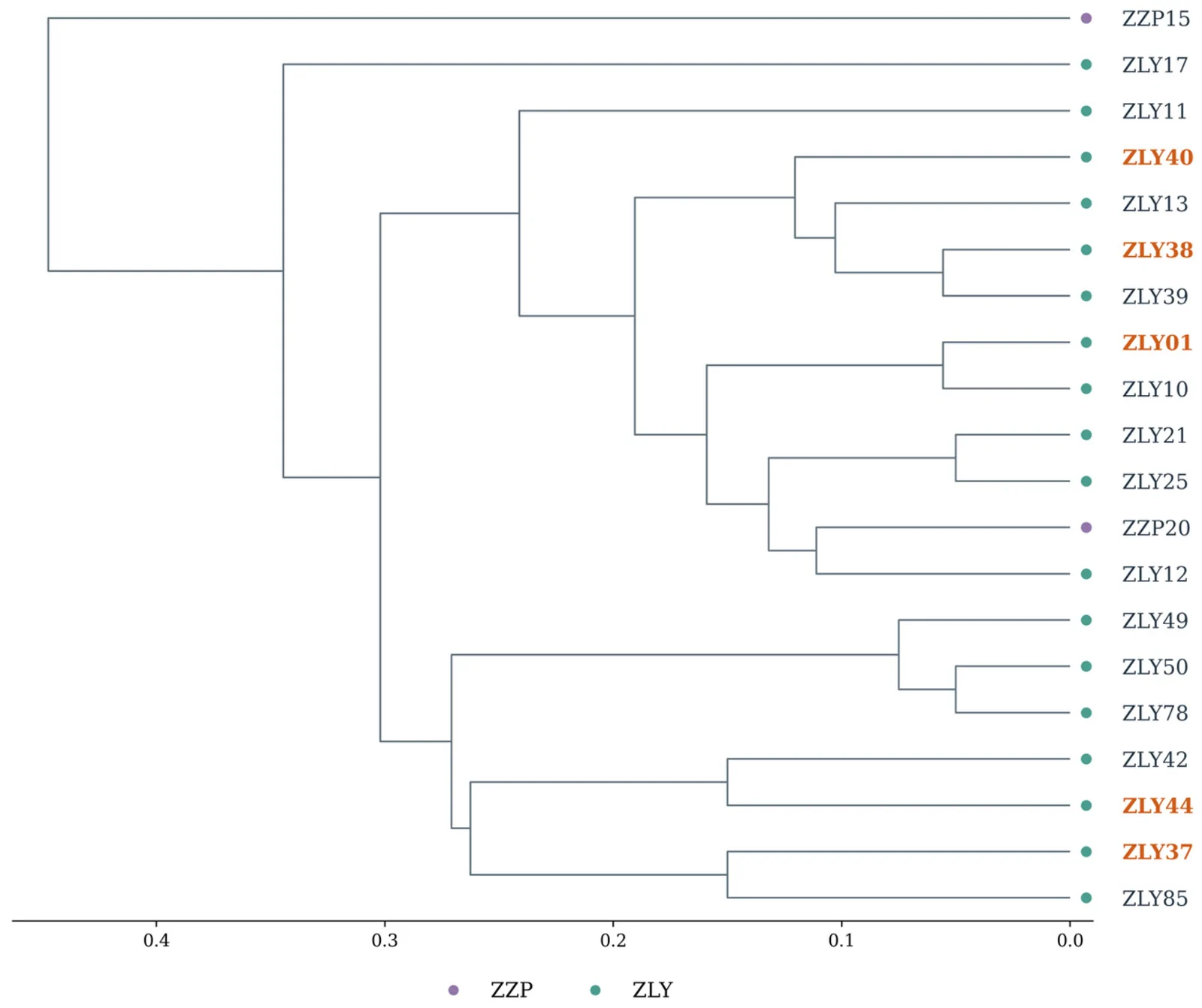
UPGMA dendrogram of the 20 individuals based on SSR profiles. Purple and teal circles beside the tip labels indicate individuals from the Germplasm Resource Nursery (ZZP) and the *Calligonum* Specialized Garden (ZLY), respectively. Bold orange labels identify the five candidate elite individuals.

Fragment-profile sharing distances based on the 10 polymorphic loci ranged from 0.050 to 0.556, with a mean of 0.276. Three pairs shared the minimum distance: ZLY21/ZLY25, ZLY49/ZLY50, and ZLY50/ZLY78. ZZP15/ZLY01 and ZZP15/ZLY11 had the maximum distance. In the unweighted pair-group method with arithmetic mean (UPGMA) dendrogram, ZZP15 joined the remaining individuals at the largest linkage distance, while ZZP20 clustered with ZLY12 (Figure 5).

### 2.5. Candidate Five-Locus SSR Panel and DNA Fingerprint Construction

**Figure 6:**
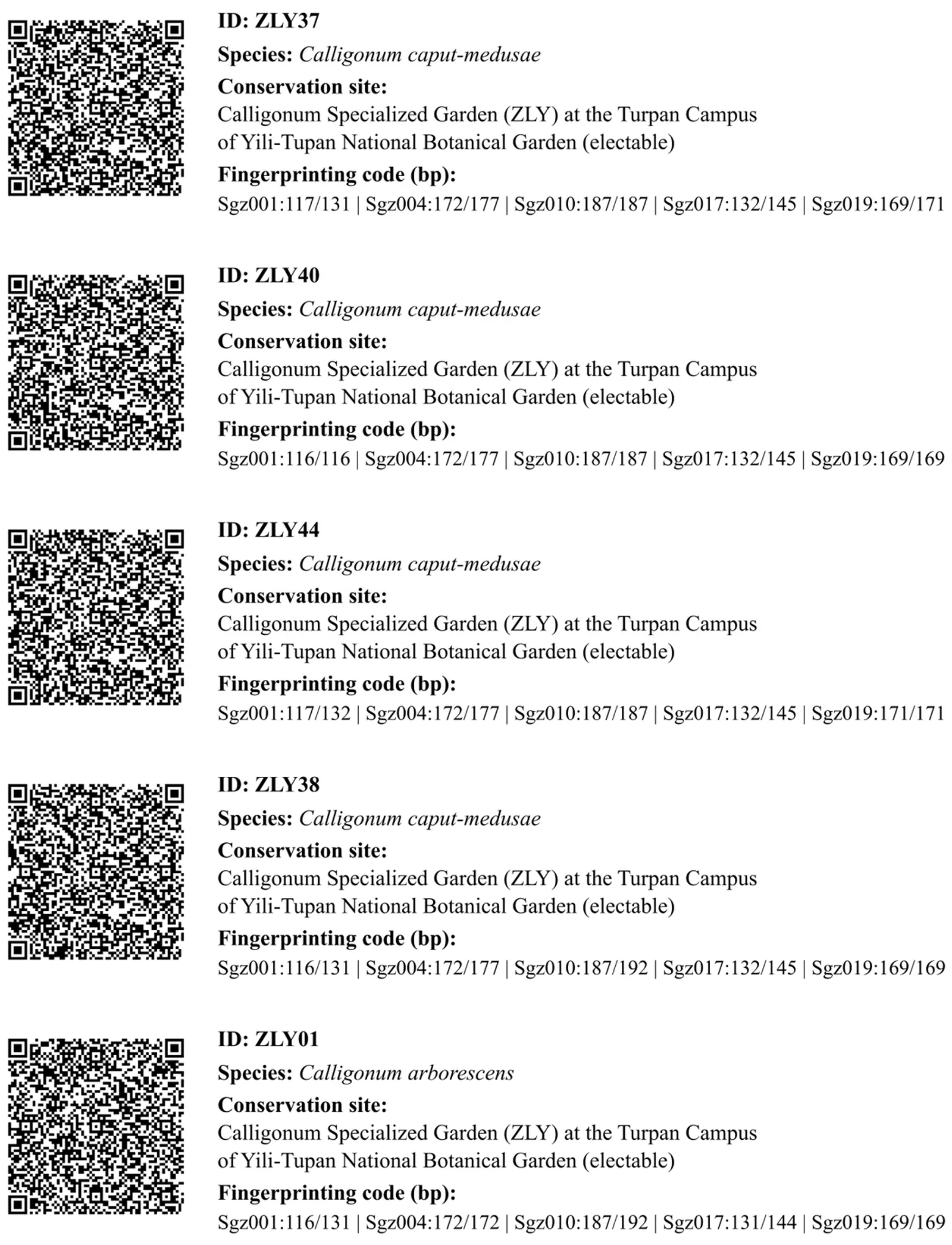
Five-locus SSR fingerprints and QR codes of the five candidate *Calligonum* sect. *Medusa* individuals, shown from top to bottom as ZLY37, ZLY40, ZLY44, ZLY38, and ZLY01. Species names are displayed beside the codes; QR payloads contain the ID, species, conservation site, and fingerprint string. Loci are ordered as Sgz001, Sgz004, Sgz010, Sgz017, and Sgz019; slashes separate the two recorded fragment sizes (bp), and vertical bars separate loci.

The combined profiles of the 10 polymorphic loci distinguished all 20 assayed individuals. Exhaustive evaluation of subsets of these loci identified a candidate five-locus panel (Sgz001, Sgz004, Sgz010, Sgz017, and Sgz019) that retained complete discrimination within this dataset (Figure S1). No one- to four-locus combination achieved complete discrimination; all five selected loci had complete profiles. Removal of any one locus from this panel resulted in at least one indistinguishable pair, indicating complementary discriminatory information among the five loci. The panel was selected and evaluated using the same 20 individuals; its discriminatory performance in independent samples remains untested. Based on this candidate panel, individual-specific multilocus fingerprint strings were generated for ZLY37, ZLY40, ZLY44, ZLY38, and ZLY01 and encoded as QR codes to facilitate identity verification and traceability (Figure 6 and Table S7).

## 3. Discussion

### 3.1. Trait Combinations of Candidate Elite Individuals and Their Ecological Relevance

Five candidate elite individuals (ZLY37, ZLY40, ZLY44, ZLY38, and ZLY01) were selected from 121 *Calligonum* sect. *Medusa* plants. Differences in time since planting, introduction origin, propagation method, and establishment history may have contributed to the observed phenotypic variation. The resulting rankings describe phenotypic performance within the present collection and do not separate genetic effects from differences in growing history. The candidates’ high D values were not attributable to extreme expression of any single trait but reflected different combinations of structural, water-status, and physiological traits. ZLY37 performed strongly on PC1, which mainly represented plant size and assimilating-branch growth, whereas ZLY01 and ZLY40 had relatively high PC2 scores, which were associated mainly with soluble sugar, midday water potential, and basal diameter. These patterns indicate that high comprehensive rankings can arise from different combinations of favorable traits rather than from a single common phenotype. Studies of woody plant breeding material in *Pinus yunnanensis*, *Fraxinus mandshurica*, and *Tilia amurensis* have similarly demonstrated the value of integrating multiple target traits when identifying candidate elite individuals [16,17,18]. Multitrait evaluation is therefore particularly appropriate for *Calligonum* germplasm intended for desert ecological restoration, where desirable material must combine structural development with physiological performance rather than maximize a single trait.

Covariation among PH, CW, BD, ABD, and ABL indicated that plant size, crown expansion, and assimilating-branch growth constituted the main structural dimension of variation. Plant height, crown width, and branch architecture can influence near-surface airflow and aeolian sand transport [9,10], supporting their relevance to ecological protection. However, PH and CW describe overall dimensions, whereas BrD measures live-branch occupancy and PFI describes crown continuity and loss. Of the six correlations between PH, CW, and ABL and the two crown-condition scores, only CW–PFI reached significance after Benjamini–Hochberg adjustment, and this association was weak (r = 0.25, q < 0.05; Figure 2). This weak association does not establish crown width as a substitute for direct assessment of crown integrity. Plant dimensions, branch density, and crown integrity should therefore be evaluated together. A larger crown need not be denser or more intact: ZLY01 combined a PH of 350 cm and CW of 306 cm with BrD and PFI scores of 3 and 2. ABL measures current-year branch extension, whereas regeneration involves seedling establishment and vegetative renewal under sand burial and erosion [5,6]. Seedling establishment and vegetative renewal were not measured in this study; consequently, regeneration capacity cannot be inferred from ABL or its correlations with other traits.

Physiological traits require more cautious interpretation. In a controlled experiment with *C. mongolicum* seedlings, water stress increased soluble sugar, proline, and MDA contents [20], whereas *C. leucocladum* plants in an arid low-salt habitat had higher soluble sugar and MDA but lower proline than those in a moist low-salt habitat [33]. These context-dependent responses indicate that physiological measurements can reflect both adaptive adjustment and the degree of stress experienced by plants. Candidate selection should therefore evaluate structural and physiological traits jointly rather than maximize any physiological indicator in isolation.

### 3.2. Multitrait Evaluation, Reduced Screening Indicators, and Preliminary Thresholds

The five retained PCs explained 68.40% of the total trait variation and integrated complementary information on plant structure, water status, and biochemical characteristics. PCA–membership function approaches are commonly used to integrate correlated traits in germplasm evaluation [16,17,34]. D therefore represents relative performance under the specified trait directions and sample-based scaling.

In the present study, the reduced model retained CW, Ψpd, PH, SS, PFI, and ABL and explained 94.89% of the variation in D while reproducing the same five highest-ranked candidates. Accordingly, the six-trait model should be interpreted as a practical approximation of the full D-based evaluation using a smaller set of directly measurable indicators. Stepwise selection and evaluation in the same dataset can produce optimistic fit and unstable predictor sets [35]; performance on additional material remains to be assessed. The retained indicators represent complementary aspects of plant performance. CW and PH reflect overall structural development, PFI describes crown integrity, ABL reflects assimilating-branch development, Ψpd indicates plant water status, and SS is associated with osmotic adjustment under water stress [5,6,7,8,9,10,19,20,36]. For each of these six indicators, the minimum observed among the five candidates was used as a preliminary lower reference threshold for joint screening within the current collection.

The present evaluation provides indirect screening based on morphological, physiological, and biochemical measurements taken during a single study period from the living collection. Differences in time since planting, size at establishment, and the developmental state of propagated material may influence subsequent plant dimensions [37]. Differences in growth performance have also been reported among *C. mongolicum* stands of different ages and in different habitats [38]; these observations do not establish a general age threshold for plant vigor. All five candidates were planted in 1977 and came from ZLY, which had higher mean PH, CW, and ABL than ZZP (Tables S2 and S5). The 1977 planting group also had higher mean PH and CW than the 2011 planting group and the unresolved material with planting records spanning 1972–2013 (Table S2). However, the 2011 group comprised only *C. roborowskii* from ZZP, whereas all other material came from ZLY; no identified species was sampled in both areas or both known planting-year groups. The unresolved material has only a planting interval that overlaps 1977 and cannot be treated as a single age group. Thus, these descriptive comparisons cannot separate time since planting from taxonomic composition, conservation area, propagation method, or other establishment conditions. The joint PH, CW, and ABL thresholds retained only *C. caput-medusae* and *C. arborescens* (Table S1), suggesting that common size criteria may favor taxa with larger mean PH and CW in this collection. Including physiological and biochemical traits in D does not demonstrate that this potential taxon-related size bias has been removed. Neither larger dimensions nor a higher D score demonstrates superior sand stabilization or restoration performance. Clonal trials across years and sites under comparable propagation, establishment, and management conditions are needed to assess the stability of candidate performance and screening thresholds. Field measurements of establishment, growth, regeneration, wind-speed attenuation, sediment transport, and sand accumulation would help determine whether the candidates improve restoration performance. The reduced indicator set and empirical thresholds provide an initial basis for prioritizing *Calligonum* material for subsequent testing.

### 3.3. SSR-Based Identity Verification and Traceability of Candidate Elite Individuals

The 10 polymorphic SSR loci provided sufficient profile variation for individual discrimination among the 20 assayed individuals, with a mean PIC of 0.357. Because ploidy was not independently established, locus-level SSR statistics were treated as descriptive summaries under the adopted binned-profile coding, whereas the marker data were used primarily for individual discrimination and pairwise relationship analysis. The candidate five-locus panel comprising Sgz001, Sgz004, Sgz010, Sgz017, and Sgz019 distinguished all 20 individuals examined, including the five candidate elite individuals. Although Sgz004 had relatively low single-locus DP, its removal resulted in at least one indistinguishable pair, indicating that multilocus discrimination depended on complementary information among loci rather than simply on selecting markers with the highest PIC or DP. Reduced SSR panels have been widely used for cultivar and clone identification [29,39]. In the present study, five-locus fingerprint records were generated for the five candidate elite individuals and encoded in QR codes to facilitate identity tracking during subsequent propagation, evaluation, and germplasm management [29,40]. In *Calligonum polygonoides*, a micropropagation protocol has also been evaluated using ISSR and SCoT markers to assess the genetic homogeneity of regenerated plants [41].

The three minimum-distance pairs differed at only one of the ten loci, which may reflect shared ancestry or limited marker resolution. The prevalence of cutting propagation in ZLY also raises the possibility of repeated clonal material, but the observed profile differences do not establish clonal identity. Replicate genotyping would help assess scoring error [28], and half-sib relationships would require pedigree information or an appropriate kinship analysis.

The five-locus panel was selected and evaluated using the same 20 individuals, so its complete discrimination represents in-sample performance and requires validation across the full collection and additional closely related *Calligonum* germplasm. Maintaining documented and traceable identities is an important component of the conservation utility of living collections [42,43]. Combined with the multitrait screening framework, molecular fingerprinting may support germplasm management, standardized propagation, cultivar development, and the use of selected *Calligonum* material in desert ecological restoration after further validation.

## 4. Materials and Methods

### 4.1. Study Site and Plant Materials

The study was conducted from June to July 2026 at the Turpan Campus of Yili-Tupan National Botanical Garden (electable), China (42.940° N, 89.184° E; approximately −105 m a.s.l.). The site has a temperate continental desert climate, with a mean annual temperature of 13.9 °C, mean annual precipitation of 16.4 mm, annual evaporation of approximately 3000 mm, and a recorded extreme maximum air temperature of 49.6 °C; summer sand-surface temperatures can exceed 80 °C [44].

The plant material comprised 121 individuals of *Calligonum* sect. *Medusa* maintained ex situ at the Turpan Campus. At assessment, all plants were fully developed, with well-expressed plant architecture and growth characteristics, and no obvious signs of whole-plant senescence were observed. For operational screening, the individuals were evaluated together under a common framework, with recorded taxonomic assignments retained for descriptive comparisons (Tables S1 and S5). No individuals showed obvious pest or disease symptoms or severe recent mechanical damage. The material included 23 individuals from the *Calligonum* Germplasm Resource Nursery (ZZP) and 98 from the *Calligonum* Specialized Garden (ZLY) at the Turpan Campus of Yili-Tupan National Botanical Garden (electable) (Figure 1). The recorded taxonomic assignments include 23 *C. roborowskii*, 22 *C. caput-medusae*, 19 *C. arborescens*, five *C. ebinuricum*, and five *C. pumilum* [45] (Table S5). The remaining 47 plants were identified only to sect. *Medusa* and may include different species. All sampled *C. roborowskii* were from ZZP; the other named species and unresolved material were sampled only in ZLY.

The ZZP was established in 2011, covers approximately 1 ha, and mainly contains seed-propagated material from different taxa and geographic origins. ZLY material was introduced successively from northwestern China and Central Asia between 1972 and 2013 and was propagated primarily by cuttings. The two conservation areas share the same general climatic setting and routine management, including one winter irrigation per year and no supplemental irrigation during the remainder of the year. The sampled plants had the following recorded planting histories (Table S5): the 23 *C. roborowskii* individuals were planted in 2011, and the 51 individuals belonging to the other four identified species were planted in 1977. The 47 individuals identified only to sect. *Medusa* were planted between 1972 and 2013; individual planting years were unavailable for this material. These records describe planting years or an interval, rather than precise plant ages from germination or vegetative propagation. The unresolved material was therefore summarized as a separate planting-record category, not as a single age group. Comparisons by conservation area and planting-record category are descriptive because planting history, taxonomic composition, and conservation area are confounded.

### 4.2. Measurement of Restoration-Oriented Traits

#### 4.2.1. Morphological Traits

Plant height (PH, cm), crown width (CW, cm), basal diameter (BD, mm), branch density (BrD), plant-form integrity (PFI), assimilating-branch diameter (ABD, mm), and assimilating-branch length (ABL, cm) were measured. PH was the vertical distance from the soil surface to the highest point of the crown. CW was calculated as the mean of the maximum east–west and north–south crown diameters, and BD was measured at the root collar using a digital caliper (Mitutoyo USA, Aurora, IL, USA). Three healthy current-year assimilating branches at comparable developmental stages were randomly selected from each individual; their diameters and lengths were measured and averaged. BrD and PFI were evaluated on predefined ordinal scales from 1 to 5. BrD was scored according to live-branch occupancy within the crown outline: 1, ≤20%; 2, >20% to 40%; 3, >40% to 60%; 4, >60% to 80%; and 5, >80%. PFI was scored according to crown continuity and crown loss associated with branch dieback: 1, >50% crown loss; 2, >30% to 50%; 3, >15% to 30%; 4, 5% to 15%; and 5, <5%. Each plant was viewed from multiple directions and independently scored by two trained observers using the same criteria. The mean of the two observers’ scores was used as the individual-level value. The scoring protocol specified joint reassessment when scores differed by more than one grade. Interobserver agreement was assessed separately for BrD and PFI using the exact agreement rate and linearly weighted Cohen’s *κ* [46]. Linear agreement weights were defined as 1 − |*i* − *j*|/4 for grades *i* and *j* on the 1–5 scale. The paired scores, cross-tabulations, and agreement calculations are provided in Table S3.

#### 4.2.2. Physiological and Biochemical Traits

Equal amounts of healthy, current-year assimilating branches at similar developmental stages were collected from the east, south, west, and north sides of each crown and pooled into one individual-level sample. Samples were frozen immediately in liquid nitrogen (Urumqi Hengsheng Dafei Trading Co., Ltd., Urumqi, China) and stored at −80 °C until biochemical analysis.

Total chlorophyll content (Chl, mg g^−1^), free proline content (Pro, μg g^−1^), soluble sugar content (SS, mg g^−1^), and malondialdehyde content (MDA, nmol g^−1^) were determined using microassay kits supplied by Sangon Biotech (Shanghai) Co., Ltd. (Shanghai, China). All samples were processed under identical quality-control conditions according to the manufacturer’s instructions. Predawn water potential (Ψpd, MPa) was measured 30–60 min before sunrise, and midday water potential (Ψmd, MPa) was measured within 1 h before or after solar noon. Two healthy current-year assimilating branches at similar developmental stages were excised from each individual, sealed immediately to minimize water loss, and measured using a WP4-T dewpoint water-potential meter (Decagon Devices, Pullman, WA, USA). The mean of two readings at each sampling period was used as the individual-level value.

### 4.3. Comprehensive Evaluation and Derivation of Preliminary Screening Thresholds

#### 4.3.1. Descriptive Statistics and Correlation Analysis

For each trait, the mean, standard deviation, minimum, maximum, and coefficient of variation (*CV*) were calculated. *CV* was calculated as follows:

$$CV=\frac{s}{\left|\bar{x}\right|}\times 100\%$$

where s and $\left|\bar{x}\right|$ are the sample standard deviation and the absolute sample mean, respectively. Linear relationships among the 13 traits were evaluated using Pearson’s correlation coefficient. Scatterplots and marginal distributions were inspected before analysis, and *p* values from the 78 pairwise tests were adjusted using the Benjamini–Hochberg procedure [30]. Means and sample SDs were also summarized for all 13 traits by recorded species, with unresolved material described separately, and for the seven morphological traits by conservation area and planting-record category (2011, 1977, and the unresolved 1972–2013 interval; Tables S1 and S2).

#### 4.3.2. PCA–Membership Function Evaluation and Hierarchical Clustering

PH, CW, BD, BrD, PFI, ABD, ABL, Chl, Pro, SS, Ψpd, and Ψmd were treated as positive-direction traits, whereas MDA was treated as a negative-direction trait.

(1) $$\mathrm{Positive}\text{-}\mathrm{direction}\;\mathrm{traits}:\;Z_{ik}=\frac{X_{ik}-{\bar{X}}_{k}}{s_{k}}$$

(2) $$\mathrm{Negative}\text{-}\mathrm{direction}\;\mathrm{trait}:\;Z_{ik}=\frac{{\bar{X}}_{k}-X_{ik}}{s_{k}}$$

where *X_ik_* is the observed value of trait k for individual i, and ${\bar{X}}_{k}$ and *s*_k_ are the mean and sample standard deviation of trait k, respectively. Unrotated PCA was performed on the Pearson correlation matrix of the directionally aligned standardized data [31]. PCs with eigenvalues *λ_j_* > 1 were retained according to the Kaiser criterion [32]. To make principal-component direction reproducible, each retained eigenvector was oriented so that the sum of its loadings across the directionally aligned traits was positive; if this sum was negative, both the eigenvector and its scores were multiplied by −1. Let *a*_j_ denote the resulting loading vector of PC *j.* The standardized PC score *F_ij_* used in the membership-function analysis was calculated as follows:

(3) $$F_{ij}=\frac{Z_{i}a_{j}}{\sqrt{\lambda_{j}}}\;\;$$

Equation (3) is equivalent to multiplying the standardized data matrix by the corresponding unit eigenvector. Following the PCA–membership function approach [47], the membership value (*U_ij_*), PC weight (*W_j_*), and relative composite score (*D*_i_) were calculated as follows:

(4) $$U_{ij}=\frac{F_{ij}-F_{j,\min}}{F_{j,\max}-F_{j,\min}}$$

(5) $$W_{j}=\frac{P_{j}}{\sum P_{j}}$$

(6) $$D_{i}=\sum (W_{j}\;\times \;U_{ij})$$

In Equations (3)–(6), *F_ij_* is the score of individual i on retained PC *j; F_j_*,min and *Fj*,max are the minimum and maximum scores for that PC; *Pj* is its percentage of variance explained; and m is the number of retained PCs. *D* is bounded by 0 and 1 and represents a relative composite score under the predefined trait directions, PC weights, and sample-based membership scaling. Ward’s minimum-variance hierarchical clustering was applied to *D* [48]. Mean profiles of all 13 traits were summarized for the six *D* groups (Table S6).

#### 4.3.3. Stepwise Regression and Derivation of a Reduced Screening Model

Stepwise ordinary least-squares regression was performed with *D* as the response variable and the 13 directionally standardized traits as candidate predictors. Variables entered the model at *p* ≤ 0.05 and were removed at *p* ≥ 0.10. Because *D* was derived from the same trait set, regression was used to obtain a parsimonious approximation of the full *D*-based evaluation rather than an independent predictive model. The six-predictor model was selected by considering *R^2^*, adjusted *R^2^*, standard error of the estimate, incremental *R^2^*, and parsimony. Agreement between observed and fitted *D* values was assessed using Pearson and Spearman correlations and by comparing the highest-ranked individuals. Unstandardized and standardized coefficients and variance inflation factors (VIFs) were calculated. For the six retained indicators, the minimum value observed among the five candidate elite individuals was used as a preliminary lower reference threshold in the original measurement units. Threshold calculations used unrounded values, and their joint application to the 121 individuals was treated as an internal consistency check. Threshold passage was tabulated by recorded taxonomic assignment (Table S1).

#### 4.3.4. Statistical Analysis and Software

Raw data were stored in Microsoft Excel 2019 workbooks (Microsoft Corporation, Redmond, WA, USA), and all final statistical analyses were performed in Python 3.12.13. pandas 3.0.5 and NumPy 2.5.2 were used for data organization, descriptive statistics, directional standardization, PCA, membership-function analysis, and calculation of *D* [49,50]. SciPy 1.16.1 was used for Pearson correlation analysis and Ward’s hierarchical clustering [51]. statsmodels 0.14.5 was used for Benjamini–Hochberg adjustment, ordinary least-squares fitting, and VIF calculation [52], and Matplotlib 3.10.6 was used for visualization [53]. Stepwise model generation, reduced-model comparison, result verification, and the interobserver agreement calculations were implemented in custom Python scripts. Statistical significance was evaluated at *q* < 0.05 for multiple-testing-adjusted correlations and at two-sided *p* < 0.05 for other tests.

### 4.4. SSR Genotyping and DNA Fingerprint Construction

#### 4.4.1. Plant Materials, DNA Extraction, and Fragment Detection

Twenty of the 121 individuals were selected for SSR genotyping, including the five candidate elite individuals and 15 comparison individuals. The comparison set was purposively selected to span the spatial distribution of plants across the two conservation areas. Young assimilating branches were collected, and genomic DNA was extracted using the cetyltrimethylammonium bromide (CTAB) method (Sigma-Aldrich, St. Louis, MO, USA) [54]. DNA integrity was assessed by electrophoresis on 1% agarose gels (Biowest, Nuaillé, France), and qualified DNA was stored at −20 °C.

Thirteen genomic SSR primer pairs previously developed for *Calligonum* were used: Sgz001, Sgz002, Sgz004, Sgz005, Sgz010, Sgz011, Sgz012, Sgz014, Sgz016, Sgz017, Sgz019, Sgz020, and Sgz023 [22]. Forward primers were labeled with FAM at the 5′ end (Table S8). The FAM-labeled primers were synthesized by Sangon Biotech (Shanghai) Co., Ltd. (Shanghai, China).

Each 25 μL PCR contained 1 μL genomic DNA, 1 μL of each forward and reverse primer (10 μM), 12.5 μL 2× EasyTaq PCR SuperMix, and 9.5 μL ddH_2_O. The amplification program was 93 °C for 3 min; 30 cycles of 94 °C for 30 s, 56 °C for 30 s, and 65 °C for 90 s; followed by a final extension at 65 °C for 5 min and holding at 4 °C [22] (Bioer Technology, Hangzhou, China). PCR products were initially checked on 1% agarose gels. Qualified products were analyzed by capillary electrophoresis at Sangon Biotech (Shanghai) Co., Ltd., and fragment sizes were scored using GeneMarker 2.2.0 (SoftGenetics, State College, PA, USA) (Table S9).

#### 4.4.2. SSR Profile Scoring and Relationship Analysis

Fragment sizes were binned into locus-specific SSR profiles using the scored capillary-electrophoresis data. Table S9 stores two fragment-size slots per individual and locus; a single distinct fragment size is repeated in both slots, a coding convention that does not establish homozygosity or allele dosage. A 0/0 record denotes an unscored individual–locus profile and was treated as missing. Missing profiles were excluded locus by locus from descriptive statistics. Because ploidy was not independently established for the 20 assayed individuals, the SSR data were used primarily for individual discrimination and pairwise relationship analysis. Locus-level Na, Ne, I, Ho, He, PIC, and DP values were therefore treated as descriptive summaries under the adopted profile coding rather than as population-level genetic diversity or structure estimates. These statistics were calculated using custom scripts in Python 3.12.13 [55,56]. Pairwise fragment-profile sharing distances were calculated from the proportion of shared fragments [57], using only loci with valid profiles in both individuals and accounting for repeated fragment sizes in the two stored slots. The resulting distance matrix was used to construct a UPGMA dendrogram using average linkage.

#### 4.4.3. Selection of a Candidate Five-Locus SSR Panel and DNA Fingerprint Construction

A Python script exhaustively evaluated all 1023 nonempty combinations of the ten retained polymorphic loci, including Sgz002 with its missing profiles. A pair was distinguishable only when at least one locus had valid profiles in both individuals and their sorted two-slot profiles differed; missingness itself was never counted as a difference. A successful panel had to distinguish all 190 pairs among the 20 individuals. The minimum was the smallest number of loci meeting this criterion. Profiles in the selected panel were concatenated in the fixed order Sgz001, Sgz004, Sgz010, Sgz017, and Sgz019 to generate multilocus SSR fingerprints (Table S7). For the five candidate elite individuals, the fingerprint information was combined with individual ID, species, and conservation site and encoded as QR codes to facilitate digital identity recording and traceability [40].

## 5. Conclusions

Multitrait evaluation of 121 *Calligonum* sect. *Medusa* individuals identified ZLY37, ZLY40, ZLY44, ZLY38, and ZLY01 as candidate elite individuals. A parsimonious six-trait model comprising crown width, predawn water potential, plant height, soluble sugar content, plant-form integrity, and assimilating-branch length explained 94.89% of the variation in D and reproduced the same five candidates, supporting these traits as practical indicators for simplified preliminary screening. Preliminary empirical reference thresholds were established for the six indicators in their original measurement units. Among 20 assayed individuals, a candidate five-locus SSR panel comprising Sgz001, Sgz004, Sgz010, Sgz017, and Sgz019 differentiated all individuals and enabled DNA fingerprint construction for the five candidate elite individuals. Together, the reduced screening model, empirical thresholds, and SSR fingerprints provide a practical framework for elite germplasm selection, identity verification, germplasm management, and subsequent cultivar development in *Calligonum* sect. *Medusa*. Further clonal, multi-year, and multi-site validation will be required before broad application in desert ecological restoration.

## Figures and Tables

**Table 1:** Descriptive statistics for 13 restoration-oriented traits measured in 121 *Calligonum* sect. *Medusa* individuals.

Trait	Unit	Mean	SD	Minimum	Maximum	CV (%)
PH	cm	234.554	64.964	152.000	483.000	27.70
CW	cm	226.376	49.210	123.500	407.000	21.74
BD	mm	70.429	37.887	40.900	212.000	53.79
BrD	score	4.339	0.748	2.000	5.000	17.24
PFI	score	3.628	0.858	2.000	5.000	23.64
ABD	mm	1.746	0.314	1.190	3.240	18.01
ABL	cm	25.266	9.719	8.300	55.170	38.47
Ψpd	MPa	−1.666	0.109	−1.876	−1.432	6.54
Ψmd	MPa	−2.671	0.326	−3.369	−2.131	12.21
MDA	nmol g^−1^	43.477	16.692	18.279	98.386	38.39
Chl	mg g^−1^	0.693	0.178	0.282	1.108	25.69
SS	mg g^−1^	15.133	4.554	6.369	26.842	30.09
Pro	μg g^−1^	102.101	49.355	33.807	302.438	48.34

Note: PH, plant height; CW, crown width; BD, basal diameter; BrD, branch density; PFI, plant-form integrity; ABD, assimilating-branch diameter; ABL, assimilating-branch length; Ψpd, predawn water potential; Ψmd, midday water potential; MDA, malondialdehyde content; Chl, total chlorophyll content; SS, soluble sugar content; Pro, free proline content; SD, standard deviation; CV, coefficient of variation.

**Table 2:** Loadings, eigenvalues, variance explained, and weights of the five retained principal components.

Trait	PC1	PC2	PC3	PC4	PC5
PH	0.793	0.285	−0.003	0.131	−0.141
CW	0.727	0.377	0.182	0.165	−0.059
BD	0.623	0.541	−0.124	0.257	−0.206
BrD	−0.125	−0.368	0.456	0.222	−0.001
PFI	0.298	−0.154	0.704	0.115	−0.134
ABD	0.682	−0.012	−0.213	−0.163	0.479
ABL	0.676	−0.197	−0.183	−0.193	0.425
Ψpd	−0.010	−0.130	0.271	0.488	0.644
Ψmd	−0.165	0.685	0.159	−0.160	0.087
MDA	0.613	−0.370	−0.034	−0.277	−0.169
Chl	0.015	−0.159	−0.529	0.684	−0.135
SS	−0.380	0.780	0.114	−0.001	0.220
Pro	−0.661	0.088	−0.249	0.016	0.123
Eigenvalue	3.557	1.978	1.286	1.045	1.026
Variance explained (%)	27.36	15.22	9.89	8.04	7.89
Cumulative variance (%)	27.36	42.58	52.47	60.51	68.40
Weight	0.400	0.222	0.145	0.118	0.115

**Table 3:** Coefficients of the selected six-trait reduced model approximating the full D-based evaluation.

Trait	B	β	t	*p*	VIF
CW	0.0506	0.497	18.129	<0.001	1.68
Ψpd	0.0291	0.286	13.449	<0.001	1.01
PH	0.0361	0.355	12.812	<0.001	1.71
SS	0.0368	0.362	15.903	<0.001	1.15
PFI	0.0192	0.188	8.386	<0.001	1.12
ABL	0.0187	0.184	7.534	<0.001	1.32

Note: B, regression coefficient applied to predictor z-scores calculated using the means and sample standard deviations of the 121 individuals; β, standardized regression coefficient; t, t statistic; *p*, nominal two-sided *p* value; VIF, variance inflation factor. Intercept = 0.4665; R^2^ = 0.9489; adjusted R^2^ = 0.9462; F(6, 114) = 352.56; *p* < 0.001; standard error of estimate = 0.0236.

**Table 4:** Preliminary empirical reference thresholds for the six reduced screening indicators of candidate elite *Calligonum* sect. *Medusa* individuals.

Category	Indicator (Unit)	Preliminary Reference Threshold
Morphological	Plant height (PH, cm)	≥297
	Crown width (CW, cm)	≥306
	Plant-form integrity (PFI, score)	≥2
	Assimilating-branch length (ABL, cm)	≥24.07
Physiological	Predawn water potential (Ψ_pd_, MPa)	≥−1.726
Biochemical	Soluble sugar content (SS, mg g^−1^)	≥16.81

**Table 5:** Genotyping success and descriptive SSR statistics for 13 loci across 20 assayed individuals.

Locus	Success (%)	Na	Ne	I	Ho	He	PIC	DP
Sgz001	100.0	4	3.213	1.229	0.750	0.689	0.627	0.715
Sgz002	80.0	2	1.438	0.483	0.000	0.305	0.258	0.305
Sgz004	100.0	2	1.995	0.692	0.950	0.499	0.374	0.095
Sgz005	5.0	1	1.000	0.000	0.000	0.000	0.000	0.000
Sgz010	100.0	4	1.891	0.899	0.600	0.471	0.433	0.685
Sgz011	100.0	3	2.100	0.792	0.950	0.524	0.410	0.185
Sgz012	100.0	1	1.000	0.000	0.000	0.000	0.000	0.000
Sgz014	100.0	2	1.105	0.199	0.000	0.095	0.090	0.095
Sgz016	100.0	2	1.105	0.199	0.000	0.095	0.090	0.095
Sgz017	100.0	5	3.704	1.402	1.000	0.730	0.684	0.645
Sgz019	100.0	3	2.020	0.857	0.100	0.505	0.442	0.565
Sgz020	100.0	1	1.000	0.000	0.000	0.000	0.000	0.000
Sgz023	100.0	2	1.220	0.325	0.000	0.180	0.164	0.180

Note: Na, observed number of alleles; Ne, effective number of alleles; I, Shannon’s information index; Ho, observed heterozygosity; He, expected heterozygosity; PIC, polymorphism information content; and DP, discriminatory power. Statistics summarize the recorded profiles under two-slot coding with unknown ploidy and allele dosage; they are not population-level estimates. Missing profiles were omitted locus by locus.

## Data Availability

The data presented in this study are available on request from the corresponding authors.

## Supplementary Materials

The following supporting information can be downloaded at https://www.mdpi.com/article/10.3390/plants15203169/s1, Table S1: Trait summaries and threshold passage by recorded taxonomic assignment; Table S2: Morphological traits by conservation area and planting records; Table S3: Interobserver agreement for branch density and plant-form integrity [58]; Table S4: Standardized principal component scores, membership values, relative composite scores, and rankings of the 121 study individuals; Table S5: Recorded taxonomic assignments, planting records, and individual-level measurements of 13 traits in 121 individuals; Table S6: Descriptive trait profiles of the six D groups; Table S7: Five-locus profiles and original fingerprint strings; Table S8: Information on the 13 simple sequence repeat (SSR) primer pairs used in this study; Table S9: Binned SSR fragment-size records for 20 individuals at 13 loci; Figure S1: SSR fragment profiles of 20 individuals based on the selected five-locus panel. All supplementary items are provided in one workbook.
